# Supplementary material for: Remanufacturing end-of-life passenger car waste sheet steel into mesh sheet: A sustainability assessment
Source: PLoS One. 2021 Oct 13;16(10):e0258399. doi: 10.1371/journal.pone.0258399 (PMC8513846; doi:10.1371/journal.pone.0258399)
Supplement: Attachment — Submitted filename: Rebuttal letter PONE-D-21-18014.docx [file pone.0258399.s001.docx]

Rebuttal Letter for Manuscript PONE-D-21-18014 "Remanufacturing End-of-life Passenger Car Waste Sheet Steel into Mesh Sheet: A Sustainability Assessment"

The responses to the reviewers’ comments appear here in red text. The changes in the revised manuscript also appear in red text.

Reviewer #1: The paper "Remanufacturing End-of-life Passenger Car Waste Sheet Steel into Mesh Sheet: A Sustainability Assessment" falls within the scope of the journal "Plos One" but doesn't meet the standard quality of the paper that should be published in one prestigious journal in the current version. A lot of core elements of one well-written and performed study is missing, so the paper needs major improvements.

Thank you for the opportunity to revise the paper. I have included most of your suggestions, as described here.

Please consider the following comments.

- Clear aims, the main contributions, and novelty are missing in the abstract. The abstract should be concise with a description of the core elements of the paper.

The abstract has been modified to include these parts (Page 2, lines 15-18, 30-35)

- The paper isn't well structured. Should be restructured: 1. Introduction, 2. Literature review, 3. Methodology, 4. Results or case study with subsections, 5. Discussion, 6 Conclusion

While I did not follow the suggested section titles, the structure of the submitted paper did indeed follow the suggested format. To further follow this standard format, the second section has been renamed “Literature Review,” and “Modelling methods” was changed to “Methodology.” As the Results and Discussion section is quite short, I believe that a combined section is acceptable.

- In the introduction section the following tasks should be fulfilled: the introduction should give an overview of the field significance, and should consider the following main questions: What are the gaps in literature? What are the main aims of this article?"

Field significance: waste vehicles account for a large fraction of land fill and contain high embodied energy that has the potential to be recovered. Remanufacturing processes have the potential to recover this “waste” material and can provide a profitable business strategy. This was addressed in the original document (Introduction).
Gaps in the knowledge: There are currently few practical remanufacturing methods for waste sheet steel. Most is sent to land fill or recovered by energy-intensive smelting processes. The proposed remanufacturing process to produce mesh sheet has potential to address these problems; however, its viability and sustainability needs to be clarified. Some further discussion of this gap in the knowledge has been added to the Introduction (Page 5-6, lines 112-113).

Main aims: The aim of this study was to evaluate the sustainability of the proposed remanufacturing process. This was stated in the final paragraph of the introduction.

- Most of the current section Introduction should be moved to new-formed 2. Literature review.

I respectfully disagree with this suggestion. In its revised form, I believe that the Introduction follows the PLOS ONE guidelines and does not include an extensive discussion of the literature. Perhaps the reviewer is referring to the section named “WSS-MSS Remanufacturing”. This is a separate section to the Introduction, and while this part does indeed discuss existing literature related to the potential waste stream, it is introducing the proposed WSS-MSS process, which is a novel process proposed in this paper. Hence, I do not think that it is appropriate to include this as a literature review.

- In the Methodology section should be ensured the diagram flow with showed research.

Thank you for this good suggestion. A flowchart of the analysis method was included (Page 12, line 240)

- Why you have decided to use best-worst multi-criteria analysis. Explain. Also, should be represented each step with equations.

The best-worst multi-criteria method was chosen as such techniques can provide a more integrated assessment to reduce large fluctuations in weight values. This explanation has been included in the text (Page 20–21; Line 365-378).

The relevant equations have been added as Eq. (6)–(9) (Pages 19–20; lines 346-364).

- More relevant references newer date should be added:

1) Biswas, T. K., & Das, M. C. (2020). Selection of the barriers of supply chain management in Indian manufacturing sectors due to COVID-19 impacts. Operational Research in Engineering Sciences: Theory and Applications, 3(3), 1-12.

2) Fazlollahtabar, H., & Kazemitash, N. (2021). Green supplier selection based on the information system performance evaluation using the integrated Best-Worst Method. Facta Universitatis, Series: Mechanical Engineering.

3) Vasiljević, M., Fazlollahtabar, H., Stević, Ž., & Vesković, S. (2018). A rough multicriteria approach for evaluation of the supplier criteria in automotive industry. Decision Making: Applications in Management and Engineering, 1(1), 82-96.

Thank you for these suggestions, they have been added as references [29], [30], and [31] in the revised manuscript.

- Comparative analysis with other MCDM methods is missing.

A comparison has been added to the text, as required (page 20,21, lines 365–378).

Reviewer #2: Thanks for giving me opportunity to review this article.

topic is very novel, different and attracting for readers. abstract and introduction sections are well designed and written. methodology section is explicitly written and stated too.

Thank you for the comments. I am happy that the reviewer was able to follow the argument of the manuscript.

some recent references can be added in terms of WSS-MSS remanufacturing process.

As this is a newly proposed remanufacturing process, there is little previous literature. I have added two of my previous conference papers to the original manuscript (refs [13,14]), but to the best of my knowledge, there are no recent references. Other references in the field of machine tool remanufacturing were included as refs [17-20].

Under results section there needs to be some changes.

Equation 8 needs to be rewritten as S12=(WMM5+WTT4+WCC3+WEE2+WSS1)/5

Same changes need to be done for Eqs. (9-12).

Thank you for bringing this to my attention. I have now defined a more general equation (Eq. 10; page 25, line 457) and this was used to calculate the SI values for all scenarios, where the exponent for E, S, and C varied. These changes have been made (pages 26-27, lines 469, 473, 478, 482, 487, 491)

some different future suggestions can be added to conclusion section.

Thank you for this suggestion, I have included some future work (page 30, 560-563)

there are some typos so proofreading can be useful.

The revised manuscript has been thoroughly checked before resubmission.

**Supplementary Information Captions**

Fig 3. Flow diagram of the analysis methods applied to evaluate the remanufacturing process.

Fig. S1 WSS–MSS remanufacturing

Fig. S2 Flow chart showing the distribution of the total weight of steel in an end-of-life car between the various waste streams [2].

Fig. S3 Photographs showing representative topological discontinuities.

Fig. S4 Estimation of sustainability performance variation of WSS from different passenger car designs.

Fig. S5 Fitting of the SI variation of WSS from different passenger car designs with SI values in the upper region of Fig. S4.

Fig. S6 Fitting of the SI variation of WSS from different passenger car designs with SI values in the middle region of Fig. S4.

Fig. S7 Fitting of the SI variation of WSS from different passenger car designs with SI values in the lower region of Fig. S4.
